# Preservation, Characterization and Exploitation of Microbial Biodiversity: The Perspective of the Italian Network of Culture Collections

**DOI:** 10.3390/microorganisms7120685

**Published:** 2019-12-12

**Authors:** Luciana De Vero, Maria Beatrice Boniotti, Marilena Budroni, Pietro Buzzini, Stefano Cassanelli, Roberta Comunian, Maria Gullo, Antonio F. Logrieco, Ilaria Mannazzu, Rosario Musumeci, Iolanda Perugini, Giancarlo Perrone, Andrea Pulvirenti, Paolo Romano, Benedetta Turchetti, Giovanna Cristina Varese

**Affiliations:** 1 Unimore Microbial Culture Collection, Department of Life Sciences, University of Modena and Reggio Emilia, via Amendola 2, 42122 Reggio Emilia, Italy; stefano.cassanelli@unimore.it (S.C.); maria.gullo@unimore.it (M.G.); andrea.pulvirenti@unimore.it (A.P.); 2 Biobank of Veterinary Resources, Istituto Zooprofilattico Sperimentale della Lombardia ed Emilia Romagna, via Bianchi 9, 25124 Brescia, Italy; mariabeatrice.boniotti@izsler.it; 3 Department of Agricultural Science, University of Sassari, viale Italia 39, 07100 Sassari, Italy; mbudroni@uniss.it (M.B.); imannazzu@uniss.it (I.M.); 4 Department of Agriculture, Food and Environmental Science, University of Perugia, borgo XX Giugno, 74, I-06121 Perugia, Italy; pietro.buzzini@unipg.it (P.B.); benedetta.turchetti@unipg.it (B.T.); 5 Agris Sardegna, Agenzia regionale per la ricerca in agricoltura, Loc. Bonassai, km 18.600 SS291, 07100 Sassari, Italy; rcomunian@agrisricerca.it; 6 Institute of Sciences of Food Production (ISPA), National Research Council (CNR), Via G. Amendola 122/O, 70126 Bari, Italy; antonio.logrieco@ispa.cnr.it (A.F.L.); giancarlo.perrone@ispa.cnr.it (G.P.); 7 MicroMiB Culture Collection, Department of Medicine and Surgery, University of Milano-Bicocca, via Cadore 48, 20900 Monza, Italy; rosario.musumeci@unimib.it; 8 Department of Life Sciences and Systems Biology, University of Turin, viale Mattioli, 25, 10125 Torino, Italy; jolanda.perugini@unito.it (I.P.); cristina.varese@unito.it (G.C.V.); 9 Mass Spectrometry and Proteomics, Scientific Direction, IRCCS Ospedale Policlinico San Martino, Largo Rosanna Benzi 10, 16132 Genova, Italy; paolo.romano@hsanmartino.it

**Keywords:** biobanks, biological resource centres, biotechnology, bioeconomy

## Abstract

Microorganisms represent most of the biodiversity of living organisms in every ecological habitat. They have profound effects on the functioning of any ecosystem, and therefore on the health of our planet and of human beings. Moreover, microorganisms are the main protagonists in food, medical and biotech industries, and have several environmental applications. Accordingly, the characterization and preservation of microbial biodiversity are essential not only for the maintenance of natural ecosystems but also for research purposes and biotechnological exploitation. In this context, culture collections (CCs) and microbial biological resource centres (mBRCs) are crucial for the safeguarding and circulation of biological resources, as well as for the progress of life sciences. This review deals with the expertise and services of CCs, in particular concerning preservation and characterization of microbial resources, by pointing to the advanced approaches applied to investigate a huge reservoir of microorganisms. Data sharing and web services as well as the tight interconnection between CCs and the biotechnological industry are highlighted. In addition, guidelines and regulations related to quality management systems (QMSs), biosafety and biosecurity issues are discussed according to the perspectives of CCs and mBRCs.

## 1. Introduction

Microorganisms and their activities have pervasive, extraordinarily profound effects on the functioning of any ecosystem, and therefore on the health of our planet and of human beings [1]. Microorganisms are everywhere; they represent most of the biodiversity of living organisms in every ecological habitat. In basic sciences, they are fundamental for the understanding and interpretation of biochemical reactions and metabolic pathways in microbial ecology research in relation, for example, to global climatic changes or societal emergences.

The coined term “microbial resources” denotes taxonomically defined, physiologically well-characterized, genetically stable, authenticated, well-documented, quality-controlled and long-term preserved strains of viruses, bacteria, archaea, fungi or protists [2].

Actually, the study and the preservation of microbial diversity are recognized as essential not only for the maintenance of natural ecosystems but also for research purposes and the development of applications in the biotech and food industries.

In this context, culture collections (CCs) and microbial biological resource centres (mBRCs) play a pivotal role in the safeguarding and circulation of biological resources and are fundamental for the progress of life sciences. Both are ex-situ repositories for biodiversity and providers of useful microorganisms (living cells and their replicable parts) and related information, for research and environmental and industrial application [3,4].

CCs reach mBRC status after having implemented both the Best Practice Guidelines defined by the Organization for Economic Cooperation and Development (OECD) and certified and/or accredited quality-assurance processes, according to dedicated standard norms [5].

Thus, mBRCs operate in a quality-controlled manner [2] and fulfil the quality standards required by the industry and by the scientific community [6]. This makes mBRCs key elements in sustainable international scientific infrastructures for the development of biotechnologies and the bioeconomy as well as in facing societal challenges.

This review deals with the services and expertise of public CCs, in particular concerning the preservation and characterization of microbial resources, by pointing to the advanced approaches applied in order to investigate a huge reservoir of microorganisms, including those that still need to be disclosed and exploited. Data sharing and web services as well as the tight interconnection between CCs and the biotechnological industry are also highlighted. In addition, guidelines and regulations related to quality management systems (QMSs), biosafety and biosecurity are discussed according to the perspectives of CCs and mBRCs.

## 2. Culture Collections and Biological Research Centre Networks: the European and Italian Scenarios

Europe has played a pioneering role in the preservation of biological resources. The first public CC was created at the end of the 19^th^ century at the German University of Prague. Within a few years, numerous other important collections arose in several European countries, which currently support a high number of CCs and host the largest and most diverse mBRCs. Most of these CCs are corporate members of the European Culture Collections Organisation (ECCO, https://www.eccosite.org/) that was established in 1981 with the aim of promoting collaboration and exchange of ideas and information about all aspects of the activity of CCs. Actually, 250 European CCs are registered in the World Data Centre for Microorganisms (WDCM, http://www.wfcc.info/ccinfo/), whose collections house more than 1.2 million strains.

Similar initiatives are underway in the USA [7,8,9], Asia [10] and South America [11]. The global CC community mainly interacts through the activities of the World Federation for Culture Collections (WFCC, https://www.wfcc.info), which actually involves 789 CCs from 77 Countries.

These networking activities are fundamental to increase the quality and efficiency of the management of CCs; most of these, indeed, have to face common challenges, i.e., the adoption of appropriate quality control, safety, security and legal procedures (although the latter may differ among different countries), as well as the need for taxonomic, systematic and bioinformatic expertise. Often, these challenges must be faced with limited staff resources and an insufficient financial commitment, which makes it difficult to maintain high standards and to guarantee excellent services [2,8].

Examples of these networking activities are the Microbial Information Network Europe (MINE), Common Access to Biological Resources and Information (CABRI), European Biological Resource Centre Network (EBRCN) and the European Consortium of Microbial Resource Centres (EMbaRC), at European level, and the Global Biological Resource Centre Network (GBRCN) at global level. These networks developed strategies to transform CCs into mBRCs. They made many efforts, with some relevant results, for the establishment of a suitable accreditation system, the definition of common standards and procedures, and the implementation of integrated on-line catalogues to facilitate consultation by the stakeholders.

The most recent and important activity of collections and research institutions in Europe is MIRRI (Microbial Resource Research Infrastructure, http://www.mirri.org), which was launched in 2010 as biomedical research infrastructure within the European Strategy Forum on Research Infrastructures (ESFRI, https://www.esfri.eu/) initiative. MIRRI seeks to improve access to high quality microbial resources and to associated services and (meta) data by creating a pan-European, distributed infrastructure of CCs, mBRCs and stakeholders [4,12,13,14].

MIRRI, in November 2018, applied for its implementation in a European Research Infrastructure Consortium (ERIC) able to bring mBRC partners under a single legal umbrella supported by interested European member states. The foreseen configuration of MIRRI-ERIC currently involves 10 countries that have already signed a memorandum of understanding (Belgium, France, Greece, Italy, Latvia, Poland, Portugal, Russia, Spain and The Netherlands). They are called to provide financial support to the implementation of the Central Coordination Unit (CCU) that will serve as the executive management office, as well as, on a national level, their own national node (NN) and serve the needs of the national community. While the CCU is clearly defined at the European level by an agreement among interested countries, the NNs may follow various forms in order to adapt to the national context.

Regarding the Italian scenario, there are numerous CCs, which preserve a huge amount of biological resources that include bacteria, yeasts, filamentous fungi, microalgae, protozoa, plasmids, cyanobacteria, archaea, virus, phages and cell lines. However, many of these collections, including some hosted by universities and research centres, do not follow relevant international standards, and their databases are not always accessible on-line. In some cases, their existence is known solely through publications, conferences or just personal contacts. Some collections are constituted for special purposes or within the frame of specific projects and sometimes their maintenance is strictly dependent on research targets, resources and facilities, as well as specific interests of the scientists involved. Up to now, coordination among these collections has still been limited.

In September 2017, a joint research unit (JRU), named MIRRI-IT (Microbial Resource Research Infrastructure – Italy, http://www.mirri-it.it), was founded by the Universities of Torino, Perugia, Modena and Reggio Emilia, the San Martino University Hospital of Genoa, and the Italian National Research Council (CNR). Actually, sixteen new associated members harbouring distinct collections joined the MIRRI network afterwards (Table 1).

**Scheme 1:**
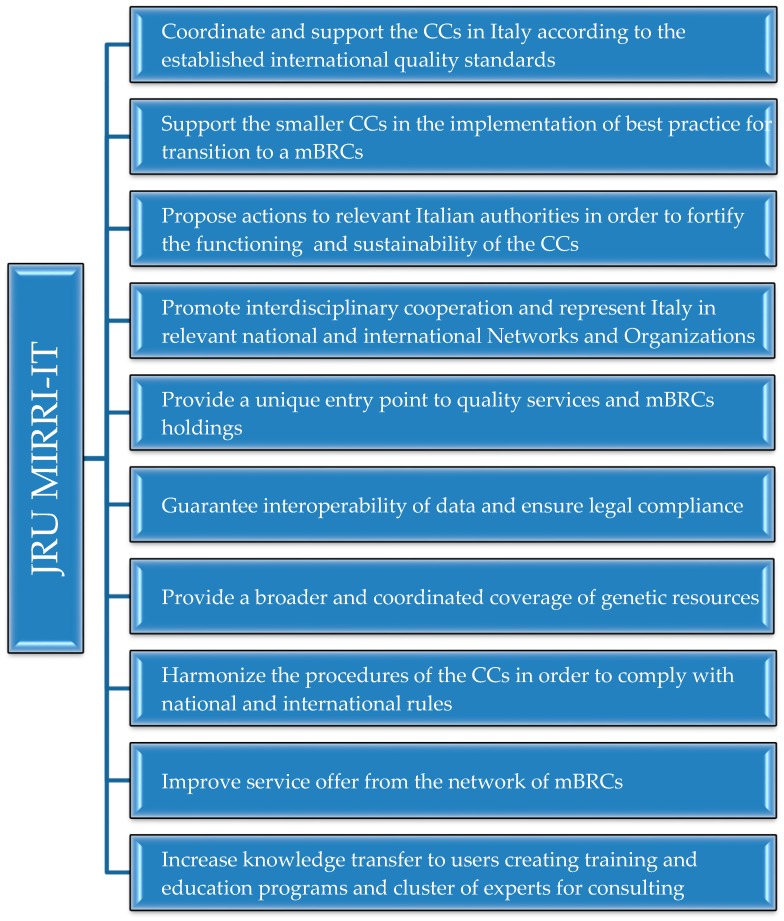
Main activities of the joint research unit MIRRI-IT.

The mission of MIRRI-IT is to overcome fragmentation in availability of resources and services offered by the Italian system of mBRCs and CCs, while enhancing the quality management system of the collections, focusing on the needs and challenges of the stakeholders interested in the biotechnological transfer of these resources. The main activities of MIRRI-IT are indicated in Scheme 1.

## 3. Services and Expertise of mBRCs

The central and crucial goals of mBRCs are the collection, preservation and distribution of microbial strains, together with the supply and exchange of related information [5]. These goals can be achieved by directing resources towards fundamental services and expertise related to (i) specific conservation and quality assurance; (ii) identification, authentication and taxonomic classification; (iii) data management and sharing.

Modern approaches in research and development of biotechnology should suggest that the cultures of microorganisms described or mentioned in publications and patent applications should be publicly available for independent studies [4]. It is thus strategic for mBRCs to keep and preserve representative and type microbial strains, as well as their potential value, for industry, medicine, agriculture and other scientific activities. Moreover, mBRCs should be able to attract investments in the biotechnological sector and support the development of the regions to which the mBRCs belong. Accordingly, safe deposit facilities and screening services are included in the core capabilities of mBRCs [15].

Moreover, mBRCs recognized as an international depositary authority (IDA) are repositories of microorganisms for patent purposes. The IDAs must comply with uniform rules and regulations including forms recommended under the international treaty signed in Budapest (Hungary) in 1977 [16].

Education and training (E&T) are other relevant services offered by mBRCs. Despite the widely recognized importance of E&T in technology transfer and innovation, multiple factors undermine the effective transfer of the know-how and expertise provided by mBRCs to academic and industrial users. However, since the demand for E&T has increased, mBRCs should rely on more updated methods and tools for improving the attractiveness of their content delivery while heightening awareness and visibility through cost-efficient and targeted advertising [1]. Therefore, playing a pivotal role in training new researchers, mBRCs may take a leading position in research infrastructures aimed at technology innovation.

### 3.1. Preservation of Biological Resources

Different techniques are widely used for ex-situ preservation and maintenance of microbial resources. These include sub-culturing on fresh media, preservation on agar beads or gelatine discs, storage in sterile soil, mineral oil, silica gel storage, spray-drying, cryopreservation, lyophilisation, liquid-drying, desiccation and vitrification [17].

According to WFCC and OECD guidelines, at least two techniques should be applied for the preservation of each microbial culture and cell stocks (master and working stocks). In addition, locally-redundant storage in separate places should be established to reduce the risk of accidental loss [18,19]. Preferably, different preservation techniques should be used to guarantee long-term maintenance and stability of the culture by reducing metabolic activity as much as possible. Cryopreservation and lyophilisation (or freeze-drying) are considered the most valuable and reliable methods for long-term maintenance of a wide range of biological resources [20]. However, they cannot be applied so effectively in a few microorganisms; complete preservation failure or reduced viability due to cell damage during the cooling and/or drying stages affects some bacteria, cyanobacteria and fungi (in particular non-sporulating cultures) or microalgae [21,22].

Therefore, preservation methods and customized protocols must be achieved for specific microorganisms by setting different parameters, e.g., suitable suspension media, cells concentration, kind of protectant and cooling rates [23,24]. Generally, freeze-drying is the preferred method for transporting and storing microbial cultures because it offers many advantages over other preservation techniques, including the total sealing of the specimen and protection from infection [25]. Validation of preservation protocols must also be carried out to ensure their reproducibility and reliability in order to assess the viability, purity, identity and stability of the preserved biological material [6]. Indeed, the composite contribution of both the adopted preservation techniques and genetic backgrounds of preserved cultures may unpredictably modify (or even lose) important technological properties [26]. This aspect may pose a serious challenge to preserving copies of genetically identical strains in different CCs [27,28]. Therefore, an extensive pre- and post-preservation characterization of cultures based on morphological, physiological, metabolic grounds and DNA analysis is required [24,29].

Usually, CCs do not supply mixed cultures, microbial consortia or microbiome samples; there are only minor exceptions relating to the provision of mycoparasites [9]. However, the MIRRI community is aware that, despite the difficulty, the preservation of complex microbial communities represents a challenge for the future. The difficulties are primarily due to the lack of an effective cryopreservation regime to optimally store both freeze tolerant and sensitive components of microbial community. CCs already face challenges related to the presence of viruses and/or bacteria within other organisms (i.e., filamentous fungi): how they can affect the phenotype of the host from a morphological and metabolic point of view [30]; how they can be quickly identified and how to characterize and conserve these microbiomes. The proper conservation of complex samples (soil, sediment, water, etc.) including their microbiomes is a future goal of the MIRRI community and, more in general, of the world microbiology community.

Indeed, several biological resources, e.g., strict anaerobes, archaea, extremophiles, fastidious bacteria, newly discovered taxa, non-sporulating fungi and viruses still require more extensive research in terms of growth optimization [29]. Furthermore, most of the microbiota occurring in some distinct niches and habitats or in extreme ecosystems such as caves, desert soils, marine water, hypersaline areas, and glaciers, cannot be cultured using standard laboratory techniques. This causes a great limitation in the exploitation of those specific microbial consortia which can be of ecological, medical or industrial interest [31].

Approximately only 1% of the actual microbial biodiversity is represented as cultured organisms, while the characteristics and functions of the remaining 99% are unknown [31]. Thus, hiring of new expertise and methodologies for cultivation of the recalcitrant microbial resources represents a crucial challenge for MIRRI CCs [32].

Usually, nutrient-rich culture media are successfully used to enhance faster-growing microorganisms at the expense of slow-growing ones. Therefore, nutrient poor media and extended incubation times (20–24 weeks) are usually employed to increase the number of oligotrophic microbes [33].

Some species can be isolated at lab scale only if co-cultivated with helper strains that release signalling molecules or specific growth-promoting factors. These molecules, when recognized, are added to the medium in order to isolate the single strain from the syntrophic species [34].

Furthermore, several advances have been made in cultivation techniques exploiting transwell plates, optical tweezers and laser microdissection, microbioreactors, or, lastly, novel diffusion bioreactors. The latter are based on a diffusion chamber that allows for the passage of necessary elements from the natural environments into the inner chamber containing the medium, which has been inoculated for enrichment [34,35,36].

Improvements regarding viruses difficult or impossible to grow on cell culture have been made with the advent of three-dimensional (3-D) cell culture methods [37,38,39,40]. In particular, “mini-intestines” or intestinal organoids provide a powerful model to propagate enteric viruses and to study viral infection, pathogenesis, and virus‒host interaction [41,42].

### 3.2. Characterization of Biological Resources

The correct characterization of preserved microorganisms is one of the main issues of the CCs that should guarantee both the authenticity and identity of the preserved microbial resources. MIRRI-ERIC have taken the first solid steps towards the development of a coordinated strategy to ensure that authentic high-quality microbial resources are available. In particular, suitable methods for the correct identification of microbial strains rely on well-defined multiloci sequence analysis combined, for some genera, with biochemical profile assessment (metabolites and/or extrolites). Concerning bacterial identification, partial or full length gene sequencing of 16S rRNA is considered as the ‘gold standard’ since it allows reconstruction of a global phylogeny [43]. For fungal identification, the internal transcribed spacer (ITS) region, has been defined as a general barcode sequence, but for a reliable taxonomic resolution of both yeasts and filamentous forms, it needs to be supported by additional gene sequencing (beta-tubulin, calmodulin, actin, elongation factor, RPB2, etc.) in relation to the specific targeted genera [44,45,46]. Genome-wide genotyping techniques such as amplified fragment length polymorphism (AFLP), pulsed-field gel electrophoresis (PFGE), and multilocus sequence typing (MLST) can be also applied. In yeast species, the use of domains 1 and 2 (D1/D2) of the LSU rRNA gene in association with the ITS region is widely accepted as a powerful tool for rapid species identification and presumptive detection of previously unknown lineages [47]. Moreover, yeast identification in clinical diagnostics is widely supported by commercial biochemical tools like API ID32C, AuxaColor, and VITEK 2 systems [48]. However, in recent years matrix assisted laser desorption ionization-time of flight mass spectrometry (MALDI-TOF MS) has been used as a potential tool for bacterial identification and diagnosis [49,50]. Other interesting automatic techniques developed for the identification and characterization of bacteria and yeast strains are Biolog and ribotyping [51,52].

Besides characterization and authentication of the microbial resources, considerable impact on new knowledge about microbial diversity comes from high-throughput sequencing techniques, which highlight, at a faster rate than traditional methods, a large number of undescribed (often unculturable) species from environmental samples.

Molecular tools, namely next-generation sequencing (NGS) technology, RNA seq, and transcriptomic and metagenomic approaches, have exponentially increased the detection of microbial biodiversity [53]. For instance, NGS has been used to uncover new fungal lineages not yet described [54]. The NGS data also increase the possibility to perform “whole genome comparison” for a better assignment of closely related microbial taxa, improving the analysis of genotypic-phenotypic relationships among different strains. In addition, the advent of NGS third generation (PacBio and Oxford Nanopore) has increased the availability of complete genomes, especially in bacteria, thus offering a valuable tool for the study of chromosomic rearrangements, repeats and translocations. It is noteworthy that, with the progressive reduction in NGS costs, it is expected that identification of novel species will be based on their entire genome [55]. In addition, genome sequences of correctly described species can be used as reliable reference to infer taxonomic lineages of so-far-uncultured microbial species in natural populations. For this reason, future taxonomic work should be conducted in silico using approaches such as GBDP (Genome Blast Distance Phylogeny) [56,57].

Therefore, mBRCs will play a crucial role in the coordination of sequencing programs by providing standardized and high-quality DNA of existing type strains. Other far-reaching advances evolved from NGS DNA sequencing along with new high-throughput phenotyping technologies are based on the use of large scale approaches to inspect how genetic instructions from a single gene or even the whole genome translate into the full set of phenotypic traits of an organism, namely “phenomics” [58]. The area of phenomics is the acquisition of high-dimensional phenotypic data on an organism-wide scale; for microorganisms, it could be performed through phenotype microarrays (PMs), or using Biolog substrate utilization data or an additional large-scale phenotypic monitoring system. However, phenomics is often hampered by the lack of good bioinformatics tools able to extract, organize in searchable databases and compute these data. Recently, the microbial phenomics information extractor (MicroPIE) was developed for extracting information from prokaryotic taxonomic and phenotypic descriptors [59]. In this respect, it is critical that the community and especially the mBRCs must take an active role in collecting, organizing and maintaining updated genotypic and phenomics data deemed worthy of long-term storage.

Therefore, ideally, mBRC bionformatic facilities should implement in-house databases to host the output from automated genome annotation pipelines and phenomics platforms along with software for basic genomic/phenotypic comparison analysis. Nevertheless, the ability to integrate local inquiry with those from publicly accessible bioinformatics platforms is advisable. Many bioinformatic platforms have been developed in the last decade, containing multidisciplinary data collected from public sequence data archives and enriched by direct submissions of external scientists. One of the most complete and integrated platforms is the Integrated Microbial Genomes & Microbiomes system v.5.0 (IMG/M, https://img.jgi.doe.gov/m/); it contains annotated datasets categorized into archaea, bacteria, eukarya, plasmids, viruses, genome fragments, metagenomes, cell enrichments, single particle sorts, and metatranscriptomes. On this platform, a plethora of complex comparisons and analyses of genomes, metagenomes and metatranscriptomes can be run by a registered user [60].

Networking among mBRCs themselves, with these platforms, will be fundamental in the future to harmonize the information through ambiguity and redundancy removal.

These demands come within the much wider context of the effort in management of digital data, so-called “big data”. Therefore, it is important, and the MIRRI project is addressing this, to revisit and provide systems architecture that can cope with big data characteristics with the aim both to hide accessing and managing data complexity and fulfil application requirements [61].

### 3.3. Databases and Data Sharing

Besides the issue related to big data management, data management itself has become an urgent and fundamental issue. Indeed, the need for proper data management procedures has been a well-known issue for mBRCs for decades. In this respect, the databases held by mBRCs with information on cultured strains need efficient and well-adapted data infrastructures. A greater standardization of such infrastructures is requested, since they are still mostly inhomogeneous, although often making reference to information for which data have been described already in the past. The level of computerization varies between mBRCs, ranging from data managed by use of relational databases to simple spreadsheet software, and this makes standardization efforts difficult. It is also worth mentioning that these culture collection information systems vary from simple collection of identification information to complex analysis platforms including experimental data, able to perform some simple bioinformatic analyses.

Current databases mainly include information on the resources (identification, molecular characterization, physio-chemical characterization), their history (provenance, acquisition, etc.) and their practical management (aliquots, storage, etc.). These databases are the starting point for the production of catalogues that can be offered on-line and made searchable or distributed, e.g., by allowing users to download them. The main objectives of these databases are the internal management of resources and the presentation of resources to potential customers for acquisition.

#### 3.3.1. Data Modelling and Interoperability

Data modelling is fundamental for every database development. It consists of the initial and detailed definition of all the information to be included in the database, their appropriate types and formats and of the logical inter-relationships between them. Such definitions must specify single data, their types (e.g., floating number, free or encoded text, date, enumeration), associated values and insertion and validation methods. Also fundamental is the definition of relations between data and additional information related to the management of data, so called metadata, which include, e.g., information on the data acquisition procedure, information on the person who carried out the insertion/update of data, and other management data.

For CCs, such data modelling has never been raised to a standard level, even if many standards were developed for the definition of information to be included in a collection database for a proper identification and characterization of resources effort [19,62,63,64]. In addition, a new ISO standard is being developed by the Biotechnology Technical Committee (TC 276) on “Specification on data management and publication in microbial resource centres” (https://www.iso.org/standard/71384.html).

As a result of lack of standard data models, information cannot be easily exchanged. Collections’ databases should not be considered as standalone information systems, but they must interact with other bioinformatics systems, with the aim of achieving a global view of data available on strains, improving access to collections’ databases and including detailed information on strains from bioinformatics databases, e.g., experimental results.

Integration of databases mainly consists of identifying links among existing information, adopting common formats for data interchange and implementing applications-driven data interchange [65]. Overall, mBRC information systems should allow access through integration software, such as workflow management systems.

#### 3.3.2. Recommendations for Collections

Some recommendations can be provided to CCs for improving the quality of their databases and allowing the integration of their contents with other life sciences information sources on the basis of the above considerations.

A special effort must be made for designing the data model and the procedures for data management. The contents of the catalogue must be revised on the basis of shared data sets and standards. It is essential that the information is encoded according to existing taxonomies and databases, e.g., for scientific names. In order to allow the establishment of bidirectional links with bioinformatic databases, it is necessary to identify all reference databases (e.g., taxonomy, chemical compounds, molecular pathways) and include in the collection database the identifiers of all referenced information.

Due to the special knowledge and expertise of the community of collections’ curators, an effort should be made to support the definition of standard formats for the information that is specific for the description of all relevant organism types. Pushing for the widespread awareness of such formats and for their adoption by managers of bioinformatics databases and tools would then be needed to make appropriate description of resources in external information sources possible.

Finally, the adoption of standard formats for delivering information and the implementation of state-of-the-art programming interfaces would allow a true and effective interoperability of collection information systems with the bioinformatics environment of databases and software.

## 4. Exploitation of Biological Resources and Connection Between Industry and mBRC: A Focus on Food Applications

Microbial resources are essential in numerous practical applications, among which are the production of food and beverages, fine chemicals, enzymes, biofuels, vaccines, antimicrobials, antibiotics and heterologous proteins. They also find application as biocontrol agents of plant and animal diseases, as indicators of environmental quality and in bioremediation processes [15,66,67]. Additionally, microbes and/or their enzymes play a vital role in transforming renewable raw materials, such as biomass, residues and CO_2_, into a huge portfolio of products (Europabio, https://www.europabio.org/).

Some microalgae, for example, could reduce CO_2_ emissions, contribute to wastewater treatment, or compete with environmental microbial contaminants, thus being useful for the realization of sustainable industrial processes, and could also find application in the food industry [22].

**Figure 1:**
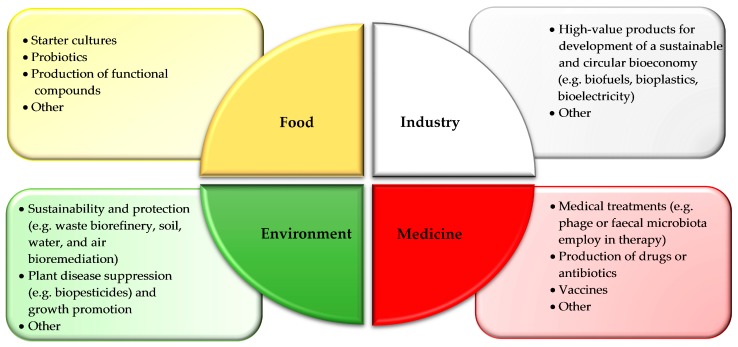
Main fields for the exploitation of microorganisms and examples of their applications.

Accordingly, microbial resources play a vital role in biotechnology innovation (Figure 1) and their exploitation in food [68,69,70], industry [71], medicine [72,73,74,75], and the environment [76,77,78] is widely reported in literature.

Indeed, the availability of certified microbial strains has to be the starting point of the selection of microorganisms exploitable in modern biotechnologies [15,79,80]. In this context, the role of mBRCs and CCs is fundamental, even the small ones, in preserving bioresources and related data to be provided for study, innovation and development [81]. In fact, the vast biodiversity preserved in CCs virtually ensures the probability of finding exceptional strains with desired properties [5].

Moreover, an increasing number of researchers are using microbial collections for large screening programs with the common intention of exploring the potential of bioresources herein preserved [15].

Therefore, industry should be persuaded to favour a long-term in-situ preservation of microbial strains by offering core support for mBRCs. Accordingly, CCs should move beyond their historical (“botanical”) context by developing commercial products and biotechnological solutions, through the screening and selection of suitable strains, and funding them through public/private investment, as well as establishing spin-off companies [5].

A global research plan, investigating microbiomes in soil, water, plants, animals, food, and human gut, would also be desirable to improve knowledge and exploitation of natural resources, share expertise and infrastructures, ensure microorganism use in research and innovation, and to promote health [82].

The worldwide food industry widely exploits microorganisms for the production of fermented food and beverages from different types of raw material, such as milk (e.g., yogurt, kefir, cheese), vegetables (e.g., pickles, sauerkraut, kimchi, tempeh, kombucha), fruit (e.g., wine, table olives), cereals (e.g., sourdough bread, beer, miso), meat (e.g., salami, sausages), and fish (e.g., fish paste) [83].

Recently, fermented food consumption has been getting a new boost, even in most developed countries, because of the health-promoting functions attributed to live microorganisms (bacteria, yeasts, and moulds) colonising these kinds of products and/or their metabolites [84]. Indeed, fermented foods can be considered as a reservoir of beneficial viable microorganisms able to survive the passage through the human gastro-intestinal tract and colonise the gut, exerting an important role in health preservation and promotion [85].

Many papers dealing with the positive effects of fermented food and related microorganisms and compounds have been published, and a comprehensive overview of health-promoting activities and compounds, per raw food matrix and related fermenting microorganisms is reported in the review published by Melini and co-workers [83].

Actually, a few companies are approaching food technologies using a relatively small number of commercial starter strains, thus causing both the detriment of the natural microbial community and the flattening of flavour and taste of fermented products [86,87].

The loss of microbial biodiversity in fermented foods could have negative results both for the products’ peculiar sensory characteristics and for human health. Indeed, consumers have shown to be increasingly attracted by peculiar traditional foods and willing to go back to their ancient flavours, as opposed to standardized ones. The large-scale use of microorganisms preserved in CCs and coming from traditional fermentation processing is, therefore, now becoming extremely important.

Indeed, it could not only allow the traditional taste of fermented foods to be recovered, but also new, natural fermented food and beverages to be obtained, able to provide health and nutrition benefits and preventing the loss of many species and strains useful for human health [82,83].

## 5. mBRC Regulations and Guidelines

The management of biological material is regulated by several national and international laws, norms and regulations concerning, among them, biosafety and biosecurity, intellectual property rights, traceability and legality of the microbial resources acquired [6,26]. Thus, the establishment of a global CC and mBRC network is central for harmonizing the exchange and the use of microbial resources and data, both for basic and applied research purposes. During the last decades, new laws and regulations have been introduced to regulate and formalize the exchange and the exploitation of genetic resources, including microorganisms, according to the convention on biological diversity [88], the Nagoya Protocol [89] and the EU Regulation N. 511/2014 [90]. The aim is to ensure the maximum benefits for academic entities and countries from which resources come from, and people worldwide, thereby contributing to the conservation and sustainable use of biodiversity. Other important normative references that the CCs must take into consideration are, for example, the regulations 90/219/EEC [91] and 98/81/EEC [92] concerning genetically modified organisms (GMOs), if they preserve this type of microorganisms within the collection, and the General Data Protection Regulation (GDPR) EU 2016/679 [93] related to the protection of personal data. The privacy regulation is particularly relevant for all CCs that preserve microorganisms isolated from human samples.

### 5.1. Quality Management System

The implementation of a quality management system (QMS) is one of the major challenges of MIRRI, which is operating actively on the design of standard guidelines [94]. Indeed, CCs are advised to start with the WFCC guidelines for the establishment and operation of collections of microorganisms. Once these principles are established, CCs should move on to apply the requirements of the OECD best practice guidelines or of specific certifications with different levels of compliance [95,96].

The International Standards Organization norm ISO 9001 (www.iso.org/iso/home/about.htm) was the first universally recognized standard management system available for many kinds of organizations (e.g., environmental, health and safety) [97]. This standard deals with the requirements that companies and organizations have to fulfil when they wish to voluntarily comply with the standard and helps them to ensure that their products and services consistently satisfy their customer needs by maintaining regulatory compliance and focusing on continuous improvement. The ISO 9001 certification is frequently used to establish confidence and improve relationships between organizations and their customers [94,98]. For these reasons, many CCs and mBRCs have introduced ISO 9001 series (ISO9001:2008 with the new version ISO9001:2015) certification as a benchmark for quality, to achieve preferred supplier status [12]. Among other general standards applied to microbiology laboratories the most common are the Good Laboratory Practice (GLP), ISO 17025, ISO 17034:2016 [24,96].

More recently, the ISO 20387, specific for biobanks, was published (https://www.iso.org/standard/67888.html). ISO 20387 has the objective to promote confidence in biobanking, defining the requirements for competence in biobank operations, including the ability to provide biological materials and associated data of appropriate quality for research and development. Several additional guideline documents will be published in the near future to help the implementation of ISO 20387, regarding the requirements for the validation and verification of methods used for the quality assurance of biological materials deposited in biobanks and the requirements for the processing, storage, transportation and data management of biological materials. In a few years, we will have a panel of documents that will guide, on a voluntary basis, those CCs/biobanks that want to be accredited and reach a high-quality status.

### 5.2. Guidelines on Biosecurity and Biosafety

A globally applicable code of conduct, specifically dedicated to biosecurity was introduced at the Seventh Review Conference at the Biological and Toxin Weapons Convention (BTWC), United Nations, Geneva, 2011 [63].

More recently, MIRRI-ERIC has proposed a biosecurity policy to support mBRCs with information on addressing biosecurity issues. The MIRRI strategy for implementation of biosecurity measures is based on the risk assessment and the establishment of an institutional biological risk policy with relevance to risk prevention. These elements lead to measures in biosecurity, which need to be implemented through harmonized procedures and monitored within a continuous improvement process [14].

To ensure biosecurity and biosafety, a global approach regarding biological risk management systems is mandatory for mBRCs. Accordingly, mBRCs must assure adequate oversight of both personnel and facilities where dangerous biological agents are located. Special precautions must be taken during collection, processing and storage of the infectious biological materials used for biobanking, and the laboratories must adopt biological safety level II or III, according to the pathogens handled [21]. Actually, any kind of activities, not only within but also outside the laboratories, for instance during the exchange of biological materials, must be taken into account.

Certainly, the degree of control that the mBRCs housing CCs should observe varies and depends on the type of biological material preserved [99]. Useful guidelines related to biosafety issues are available from the Centre for Disease Control (Biosafety in Microbiological and Biomedical Laboratories, V Edition; https://www.cdc.gov/labs/pdf/CDC-BiosafetyMicrobiologicalBiomedicalLaboratories-2009-P.PDF) and from the World Health Organization (Laboratory Biosafety Manual, II Edition; https://www.who.int/csr/resources/publications/biosafety/Labbiosafety.pdf?ua=1).

## 6. Conclusions and Future Prospects

Since microorganisms play a fundamental role in maintaining natural ecosystems and in developing valuable applications in the biotech and food industries, the loss of microbial biodiversity must be opposed and prevented from both an ecological and a socio-economical point of view. However, despite its undeniable intrinsic value, microbial biodiversity is largely ignored in debates on the conservation and management of global diversity, possibly due to a generalized misperception of the microbial world. In fact, on the one side microorganisms are invisible to the naked eye. Thus, their essential role in the functioning and health of all forms of life on our planet can be easily overlooked. On the other side, they are often perceived as agents of diseases more than fundamental resources.

CCs and mBRCs have a vital role in the preservation, characterization, management and exploitation of microbial biodiversity to address societal challenges. Moreover, by guaranteeing the distribution of high-quality microbial resources they ensure reproducibility and therefore the quality and credibility of science. In the near future, they will be in charge of the conservation of uncultivable microorganisms, microbial consortia and microbiome samples, all important challenges that must be faced in order to safeguard irreplaceable microbial resources to be delivered to future generations.

The organizations involved in mBRC networking activities at the national and supranational levels are fundamental to increase and harmonize the management of ex situ microbial repositories, for the circulation of related data and for the standardization of norms and regulations concerning biosafety and biosecurity, intellectual property, traceability and legality of the microbial resources.

Indeed, all the actors involved at the different levels in the accomplishment of these tasks have to face problems connected to limited human and economic resources. Thus, it is mandatory to stimulate the public debate and involve an increasing number of countries in this virtuous process in order to form enlarged partnerships and also for the achievement of new funding opportunities. MIRRI-ERIC can serve as a catalyst for this process at both the European and global level.

## Figures and Tables

**Table 1:** Partners and associated member of the joint research unit MIRRI-IT.

MIRRI-IT* Partners ^1^ and Associated Members ^2^	Culture Collections	Biological Resources (Main Taxa)	Isolation Sources (Category)
**Research Centres:**			
Agenzia nazionale per le nuove tecnologie, l’energia e lo sviluppo economico sostenibile (ENEA) ^2^	ENEA collection	Bacteria, Fungi, Microalgae, Microbial consortia, Virus	Contaminated sites, Hypogea and archaeological sites, Food, Lake sediments, Sea, Soil, Rhizosphere, Water
Agenzia regionale per la ricerca in agricoltura, (Agris Sardegna) ^2^	Bonassai (BNSS)	Bacteria (Actinobacteria, Bacteroidetes, Firmicutes, Proteobacteria)	Animals, Food
Consiglio Nazionale delle Ricerche (CNR) ^1^	Agro-Food Microbial Culture Collection (ITEM)	Bacteria, Filamentous fungi, Yeasts	Air, Insects, Food, Plants, Soil
	Plant Viruses Italy (PLAVIT)	Mycoviruses, Phytoplasmas, Phages Plant viruses	Fungi, Plants
IRCCS Ospedale Policlinico San Martino of Genoa ^1^	Interlab Cell Line Collection (ICLC)	Cell lines	
Istituto Nazionale Malattie Infettive (INMI) “Lazzaro Spallanzani”, Roma ^2^	INMI collection	Bacteria, Yeasts	Humans, Clinical isolates
Istituto Nazionale di Oceanografia e di Geofisica Sperimentale, Trieste (OGS) ^2^	Collection of Sea Microorganisms (COSMI)	Microalgae (predominantly Bacillariophyceae, Dinophyceae,)	Marine environments
Istituto Zooprofilattico Sperimentale della Lombardia e dell’Emilia Romagna (IZSLER) ^2^	Biobank of Veterinary Resources (BVR)	Cell lines, Bacteria, Fungi, Parasite, Virus	Animals, Feed, Food, Environments
Istituto Zooprofilattico Sperimentale delle Venezie (IZSVE) ^2^	European Union Reference Laboratory (EURL) Biobank	Viruses, Antigens, Polyclonal antisera	Animals
**Universities:**			
Sapienza University of Rome, Department of Environmental Biology ^2^	Fungal Biodiversity Laboratory (FBL)	Saprotrophic fungi	Litter, Contaminated sites, Soil, Water
University of Cagliari ^2^	DSMSP Collection	Bacteria, Moulds, Yeasts	Beverages, Environments, Food
University of Basilicata, School of Agricultural, Forestry, Food and Environmental Sciences ^2^	UNIBAS Yeast Collection (UBYC)	Yeasts	Beverages, Food
University of Genoa ^2^	Collection of DISTAV (ColD)	Bacteria, Fungi, Yeasts	Extreme environments, Human cadaver
University of Milano-Bicocca ^2^	MicroMiB collection (MicroMib)	Bacteria, Yeasts, Virus	Humans, Environments, Food
University of Modena and Reggio Emilia ^1^	Unimore Microbial Culture Collection (UMCC)	Bacteria (Lactic Acid Bacteria, Proteobacteria), Yeasts (Ascomycota)	Beverages, Food
University of Naples “Federico II”, Department of Biology ^2^	Algal Collection University Federico II (ACUF)	Cyanobacteria, Microalgae	Aeroterrestrial habitats, Environments
University of Palermo, Department of Agricultural, Food and Forest Sciences ^2^	Herbarium SAF (SAF)	Macrofungi	Ascomata, Basidiomata
University of Pavia, Department of Earth and Environmental Sciences ^2^	Amico Fungo	Filamentous Fungi, Yeasts	Soil, Contaminated sites
University of Perugia ^1^	Industrial Yeasts Collection (DBVPG)	Yeasts, Yeast-like organisms	Beverages, Environments, Food
University of Sassari ^2^	Microbial Culture Collection of University of Sassari (UNISS)	Bacteria (Firmicutes, Proteobacteria), Filamentous fungi, Yeasts (Ascomycota, Basidiomycota)	Agricultural by-products, Animals, Beverages, Environments, Food, Plants, Humans
University of Turin ^1^	Turin University Culture Collection (TUCC) including Mycotheca Universitatis Taurinenesis (MUT)	Bacteria (Firmicutes, Lactic Acid bacteria, Proteobacteria) Filamentous fungi (Ascomycota, Basidiomycota, Mucoromycota), Yeasts	Animal and human clinical Samples, Extreme environments, Food
University of Tuscia, Department of Ecological and Biological Sciences ^2^	Culture Collection of Fungi from Extreme Environments (CCFEE)	Filamentous and Meristematic fungi, Lichens, Yeasts	Monuments, Contaminated sites, Rocks, Soil

* www.mirri-it.it; ^1^ = partners of MIRRI-IT; ^2^ = associated members of MIRRI-IT.
